# Electroencephalography-demonstrated mechanisms of dexmedetomidine-mediated deepening of propofol anesthesia: an observational study

**DOI:** 10.1186/s13741-021-00213-4

**Published:** 2021-12-09

**Authors:** Lei Zhang, Kun Fang, Shengwei Tao, Liyun Deng, Hua Li, Yuanyuan Cao, Lei Wang, Fengqiong Yu, Erwei Gu

**Affiliations:** 1 Department of Anesthesiology, The First Affiliated Hospital of Anhui Medical University, Anhui Medical University, Hefei, Anhui China; 2 First School of Clinical Medicine, Anhui Medical University, Hefei, Anhui China; 3 School of Mental Health and Psychological Sciences, Anhui Medical University, Hefei, Anhui China

**Keywords:** Anesthesia monitoring, Anesthesia depth, Electroencephalogram, Dexmedetomidine, Propofol

## Abstract

**Background:**

Although dexmedetomidine (Dex) is known to reduce bispectral index (BIS) values and propofol dosage, there is little information regarding raw electroencephalography (EEG) changes related to Dex deepening of propofol general anesthesia (GA). This study investigated the Dex effects on propofol GA via analysis of EEG changes.

**Methods:**

A study cohort of 21 surgical patients (age range, 20–60 years) categorized as American Society of Anesthesiologists (ASA) class I or II was enrolled. We used time-varying spectral and bicoherence methods to compare electroencephalogram signatures 5 min before versus 10 min after intravenous Dex injection under propofol GA. The means and medians are reported with 95% confidence intervals (CIs) and inter-quartile ranges (IQRs), respectively.

**Results:**

Dex augmented the slow waves power and theta (*θ*) oscillation bicoherence peak from a mean (95% CI) of 22.1% (19.0, 25.2) to 25.2% (21.8, 28.6). Meanwhile, Dex reduced alpha (*α*) peak power and bicoherence from 3.5 dB (1.0, 6.0) and 41.5% (34.0, 49.0) to 1.7 dB (− 0.6, 4.0) and 35.4% (29.0, 41.8), respectively, while diminishing the median frequency of *α* oscillation peak values and the mean frequency of *α* peaks in bicoherence spectra from 12.0 Hz (IQR 11.2, 12.6) and 11.7 Hz (11.3, 12.2) to 11.1 Hz (IQR 10.3, 11.8) and 11.2 Hz (10.9, 11.6), respectively.

**Conclusions:**

Profound EEG changes support the supposition that Dex enhances propofol-induced GA from a moderate to a deeper state. The present findings provide a theoretical basis and reference regarding protocols aimed at reducing anesthetic/sedative dosage while maintaining sufficient depth of GA.

**Clinical trial registration:**

ChiCTR, ChiCTR1900026955. Registered on 27 October 2019

**Supplementary Information:**

The online version contains supplementary material available at 10.1186/s13741-021-00213-4.

## Introduction

Dexmedetomidine (Dex) is a selective alpha type 2 (α2) adrenoceptor agonist that has become a preferred anesthetic and sedative in operating rooms and intensive care units owing to its unusual characteristic of targeting primarily presynaptic α2 adrenergic receptors on locus coeruleus (LC) projection neurons, thereby affecting arousal without the peripheral side effects that occur with less selective agents. Under Dex sedation, patients experience less respiratory depression and, postoperatively, are more easily aroused and better able to communicate than with propofol (Hsu et al., 2004). Owing to its more limited action, Dex can be combined with other components to construct a combined action.

When administered together with propofol, Dex has been shown to reduce the dose of propofol required to produce loss of consciousness (Peden et al., 2001), suggesting that it can be used to facilitate the depth of general anesthesia (GA) achieved with propofol. Dex has been reported to reduce the propofol dose needed to suppress bispectral index (BIS) values to unconsciousness levels during GA (Dutta et al., 2019; Le Guen et al., 2014). Notably, Dutta and colleagues (Dutta et al., 2019) found that Dex reduced propofol anesthesia induction and maintenance doses by 15% and 29%, respectively. However, few studies have examined electroencephalography (EEG) signal changes during Dex-facilitated propofol GA, which may provide a theoretical basis and reference data to enable minimal drug exposure while maintaining the requisite depth of anesthesia (Dutta et al., 2001; Purdon et al., 2015a; Brown et al., 2018).

Although anesthetic dosage and BIS values could potentially be affected by hypotension and compromised cerebral perfusion, Dex does not appear to impair cerebral perfusion in healthy volunteers (Farag et al., 2017). Hence, the Dex-induced sedation-associated brain states are likely attributable to drug-specific effects on neurophysiological circuits. Dex-mediated inhibition of LC neurons and norepinephrine (NE) release (Jorm & Stamford, 1993; Nacif-Coelho et al., 1994; Nelson et al., 2003) reduces inhibitory input to the preoptic area, allowing activation of gamma-aminobutyric acid (GABA) releasing inhibitory projections from the preoptic area to the arousal center, thus causing sedation. Propofol is believed to induce sedation and unconsciousness primarily via its action on GABA_A_ (type A) receptors (Bai et al., 1999; Hemmings Jr. et al., 2005). The pharmacological effects of general anesthetic and sedative agents can be distinguished by characteristic EEG signatures that reflect the depth of anesthesia (Purdon et al., 2015a). Previous research examining frontal EEG power and coherence patterns (Akeju et al., 2014a; Akeju et al., 2014b; Xi et al., 2018) has shown that propofol-induced unconsciousness is characterized by highly coordinated frontal cortex alpha (*α*; 8–12 Hz) oscillations, delta (*δ*; 1–4 Hz) oscillations, and high-amplitude synchronous slow (0.1–1 Hz) oscillations, whereas Dex sedation presents as a combined slow-δ oscillation with sleep spindle activities, mostly in a frequency range similar to that of the *α* oscillations seen with propofol. Based on established molecular pharmacology and neural mechanism knowledge, neural network changes underlying EEG changes that occur with deepening anesthesia state can be postulated.

Propofol *α* oscillations and Dex spindles have been associated with the regulation of thalamocortical circuits (Ching et al., 2010; Supp et al., 2011). Therefore, we hypothesized that changes in GABA-regulated thalamocortical reverberant networks are involved in Dex-mediated alterations in propofol EEG and bicoherence patterns. To investigate this hypothesis, we conducted an EEG study of patients undergoing Dex-supplemented propofol GA.

## Methods

### Patient selection and anesthesia protocols

This observational study was carried out at the First Affiliated Hospital of Anhui Medical University (Hefei, Anhui, China) between November 2019 and April 2020. All patients provided informed consent. The enrolled patients were adults ranging from 20 to 60 years of age with an American Society of Anesthesiologists (ASA) class I or II physical status of who were scheduled for surgery under general anesthesia. The exclusionary criteria were a history of neurological, cardiovascular, hepatic, or metabolic disease; any reason to anticipate a difficult airway; and body weight > 130% of ideal body weight.

All patients were not given premedication. Standard noninvasive blood pressure monitoring, pulse oximetry, electrocardiography, and EEG were instituted before anesthesia induction. Propofol (2 mg kg^−1^), sufentanil (0.5 μg kg^−1^), and cisatracurium (0.2 mg kg^−1^) were injected for anesthesia induction, followed by continuous propofol infusion at a rate of 4 mg kg^−1^ per hour. After 15 min of laryngeal mask insertion, an intravenous bolus of Dex (0.8 μg kg^−1^) was administered over 10 min followed by Dex maintenance at a rate of 0.5 μg kg^−1^ per hour. For each patient, the 3-min artifact-free EEG segments of 5 min before and 10 min after Dex injection were retrospectively compared by using time-varying spectra and bicoherence analyses. During the whole process, ephedrine and atropine were used as needed to maintain systolic blood pressure above 90 mmHg and heart rate above 55 beats/min. Data collection for analysis was completed before the start of surgery.

### Data collection and EEG preprocessing

**Fig. 1:**
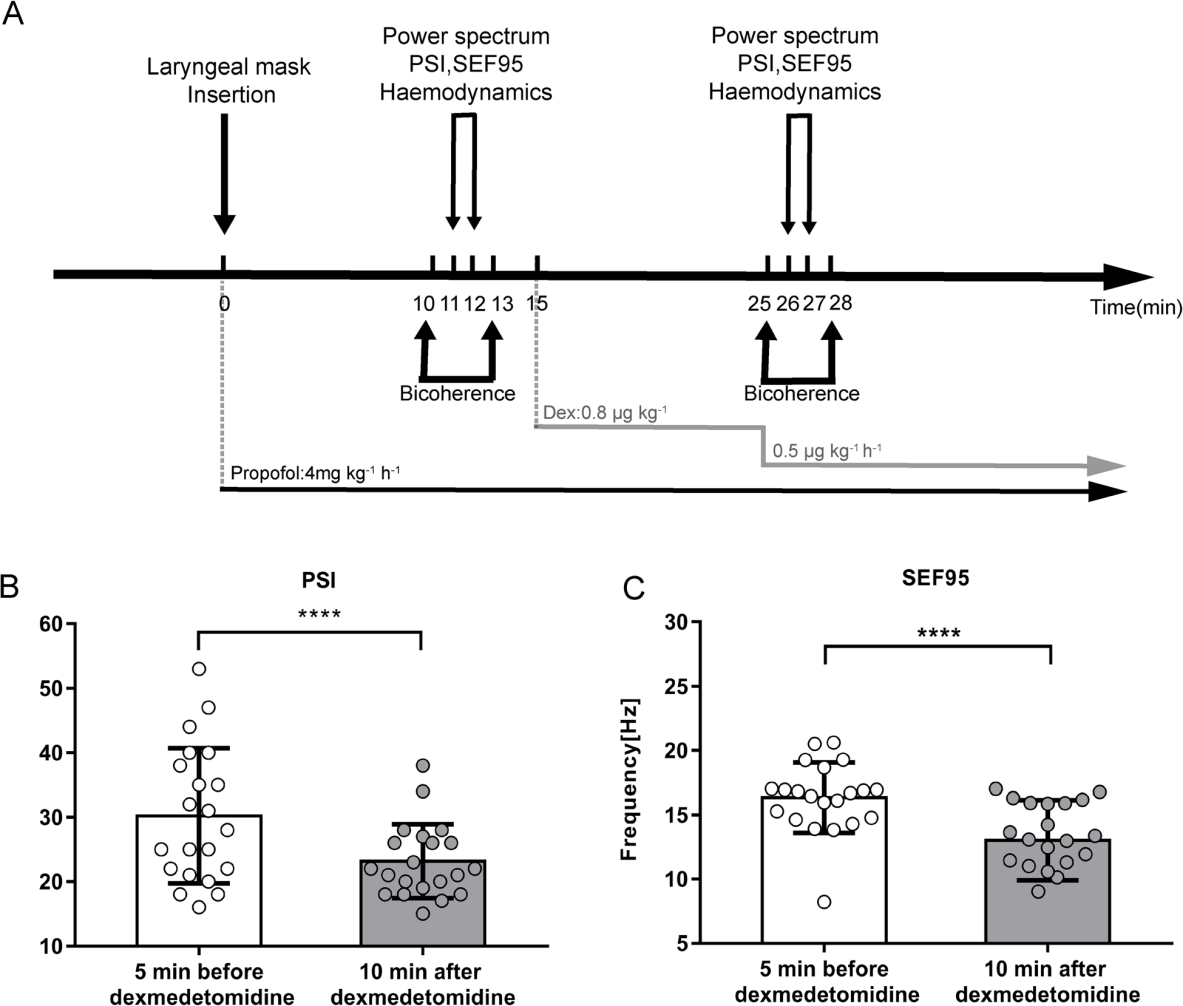
Experimental protocol and anesthesia monitoring data. **A** Experimental flow chart in the operating room. EEG data were collected for two 3-min periods, one commencing 5 min before and one 10 min after dexmedetomidine (Dex) injection during propofol anesthesia. **B** Comparison of the mean patient state index (PSI) values across the two time points. **C** Comparison of the mean spectral edge frequency 95 (SEF95) values 5 min before versus 10 min after dexmedetomidine injection. Note that both PSI and SEF95 values were markedly decreased at the second time point relative to the first, consistent with a deepening of anesthesia; ****p < 0.0001, paired *t* tests. Error bars show the standard deviations (SDs)

A summary of the experiment protocol and data collection procedure is presented in Fig. 1A. All data were collected before the surgery began. Frontal EEG data were recorded via a Sedline brain function monitor (Masimo Corporation, Irvine, CA) with a preamplifier bandwidth of 0.5–89 Hz and a 178-Hz sampling rate. All electrode impedances were maintained below 5 kΩ. The standard six-electrode Sedtrace electrode array was used, including electrodes at Fp1, Fp2, F7, and F8; a ground electrode at Fpz; and a reference electrode ~ 1 cm above Fpz.

The patient status index (PSI) and 95% spectral edge frequency (SEF) were used to monitor the depth of anesthesia, calculated from raw-EEG. Analogous to the BIS, the PSI is a dimensionless number scaled from 100 to 0, with 100 representing an awake EEG and 0 denoting complete electrical silence. SEF95 is the frequency which 95% of the power in the spectrum below this frequency. Lower values below 30 Hz usually indicate a lower level of responsiveness (Kuizenga et al., 2018).

We collected the spectral edge frequency 95 (SEF95) and patient state index (PSI) index values every minute. The raw signals were subjected to a 0.5–50-Hz band-pass filter with a linear finite impulse response filter (EEGLAB toolbox) (Tort et al., 2008). An experienced investigator identified and rejected noise and artifacts. For each patient, we analyzed two 3-min artifact-free EEG segments, one starting 5 min before dexmedetomidine injection and the second starting 10 min after dexmedetomidine injection, in Matlab to perform EEG analysis (spectral analysis and bicoherence analysis). Differences in the following parameters were also assessed between the 5 min before and 10 min after Dex infusion time periods: PSI, SEF95, *α* power peak, *α* bicoherence peak, frequencies of the *α* power and at *α* bicoherence peaks, theta (*θ*) bicoherence peak, and frequencies at the *θ* bicoherence peak.

### Spectral analysis

The EEG data were processed into power spectra, which reflect the distribution of signal power over a range of frequencies over time. We generated and analyzed power spectrograms in Chronux (a Matlab tool) using the multitaper method(Bokil et al., 2010) with the following parameters: a 2-s window length with 0-s overlap, a time-bandwidth product of three with five tapers, and a spectral resolution of 3 Hz. Group-level spectrograms were computed by taking medians across all patients at each time point. The group median spectra and 95% confidence intervals (CIs) were calculated with the bootstrap procedure for selected EEG epochs (Purdon et al., 2015b). Bootstrap spectrogram samples comprised 90 non-overlapping 2-s EEG windows drawn from the full sample of data for each subject; bootstrap spectra samples were computed by taking median across all time epochs for each subject. Then, the median bootstrap spectra samples were taken across all subjects to obtain the median bootstrap spectra estimate. We repeated this procedure 10,000 times and used the percentile method to calculate 95% CIs of the median spectra.

### Bicoherence calculation

Bicoherence, which is a normalized bispectral analysis parameter, can be used to track nonlinear regulation of neuroelectrical activity changes by quantifying the quadratic phase coupled in the thalamocortical regulation that links signals across cortical areas via a single-channel cortical electroencephalogram.

We computed bicoherence from the selected artifact-free, 3-min periods of signals (paired frequencies, 0.5–20 Hz at 0.5-Hz intervals) and represented the results as two-dimensional moving averages. We used nine bicoherence points (spaced by 0.5 Hz) to calculate the diagonal bicoherence from 1.5 to 20 Hz, as described by Araki et al. (Araki et al., 2018), then divided the 3-min-long EEG signal datasets into 360 epochs and applied the Blackman window function (2 s, 75% overlap) to each epoch. The equations used for calculating the bicoherence, represented as BIC (*f*_1_*, f*_2_), were as follows (Araki et al., 2018):

The sum of absolute triple product:

$$ \mathrm{sTP}\left({f}_1,{f}_2\right)={\sum}_{i=1}^L\left|{X}_i\left({f}_1\right){X}_i\left({f}_2\right){X_i}^{\ast}\left({f}_1+{f}_2\right)\right| $$

$$ bispectrum:B\left({f}_1,{f}_2\right)=\mid {\sum}_{i=1}^L{X}_i\left({f}_1\right){X}_i\left({f}_2\right){X_i}^{\ast}\left({f}_1+{f}_2\right)\mid $$

$$ BIC\left({f}_1,{f}_2\right)=100\frac{B\left({f}_1,{f}_2\right)}{\mathrm{sTP}\left({f}_1,{f}_2\right)} $$

*j*: epoch number

*X*_*j*_(*f*_1_): complex value calculated by Fourier transformation of the *j*th epoch

$$ {X_i}^{\ast}\left({f}_1+{f}_2\right): conjugate\ of\ {X}_i\left({f}_1+{f}_2\right) $$

The group median diagonal bicoherence values (when *f*_1_ = *f*_2_) and associated 95% CIs were calculated by the bootstrap method described in the previous subsection. Bootstrap samples were drawn from the full sample of diagonal bicoherences for each subject, then bootstrap sample medians were determined to compute median diagonal bicoherence.

### Statistical analysis

According to the previous studies (Hagihira et al., 2002) and our pre-experiment, the mean of alpha bicoherence and the SD of *α* bicoherence were approximately − 5 dB and 6 dB, respectively. A sample size of 16 achieves a statistical difference with 91% power and a significance level (alpha) of 0.050. Considering the quality of the data, we recruited 26 patients and ended up with 21 data to analyze. The median spectral differences and 95% CIs were calculated by the bootstrap method described in the previous subsection to obtain the differences between spectra at each frequency. After calculating the bootstrap spectra samples, we computed the estimated differences between experimentally compared spectra samples for each subject and took a median across all the subjects. The differences were considered significant only if the contiguous frequency bandwidth was wider than the spectral resolution (Flores et al., 2017).

Based on the results of Shapiro-Wilk normality testing, the Wilcoxon signed-rank test and paired *t* tests were used to analyze the non-parametric and parametric data parameters, respectively, in the Prism version 5.0 software (GraphPad, San Diego, CA). In the former case, the data are reported as medians with inter-quartile ranges (IQRs; 25th–75th percentile). In the latter case, the data are expressed as means with 95% CIs. In all cases, *p* < 0.05 was considered statistically significant. Inter-median and inter-mean differences were analyzed with the bootstrap method and are reported with 95% CIs.

## Results

Of the 26 enrolled patients, 5 were excluded due to poor-quality EEG (*N* = 3) or bad channels (*N* = 2) ( see details in Supplementary Fig.S1). The 21 remaining patients were included in the final analysis. Their patient characteristics are summarized in Table 1. Bradycardia (HR < 55 beats per min) occurred in 2 of 21 patients during DEX infusion.

**Table 1:** Characteristics of the present study group of patients given propofol anesthesia supplemented with dexmedetomidine

Characteristic	Mean ± SD or *N* (%)
Age, years	42 ± 10.7
Sex, no. of males	11 (52%)
Bodyweight, kg	66.6 ± 11.2
Body mass index, kg m^−2^	23.8 ± 2.9
ASA class	
I	6 (29%)
II	15 (71%)

Figure 1 shows the experimental protocol and changes of anesthesia depth monitoring indexes 5 min before and 10 min after Dex injection. We observed marked Dex injection-induced differences in the spectrogram in patients under propofol GA. Compared with baseline (5 min before injection), the PSI and SEF95 decreased significantly (indicating deeper anesthesia) from the mean values of 30.2 (25.5 to 35.0) and 16.3 Hz (15.1 to17.6 Hz) to 23.2 (20.6 to 25.8) and 13.1 Hz (11.6 to 14.4 Hz) (Fig. 1B, C; mean (95% CI), both *p* < 0.0001), with bootstrap mean differences (95% CI) of − 7.1 (− 12.1 to − 2.2) and − 3.3 Hz (− 5.1 to − 1.6 Hz), respectively.

**Fig. 2:**
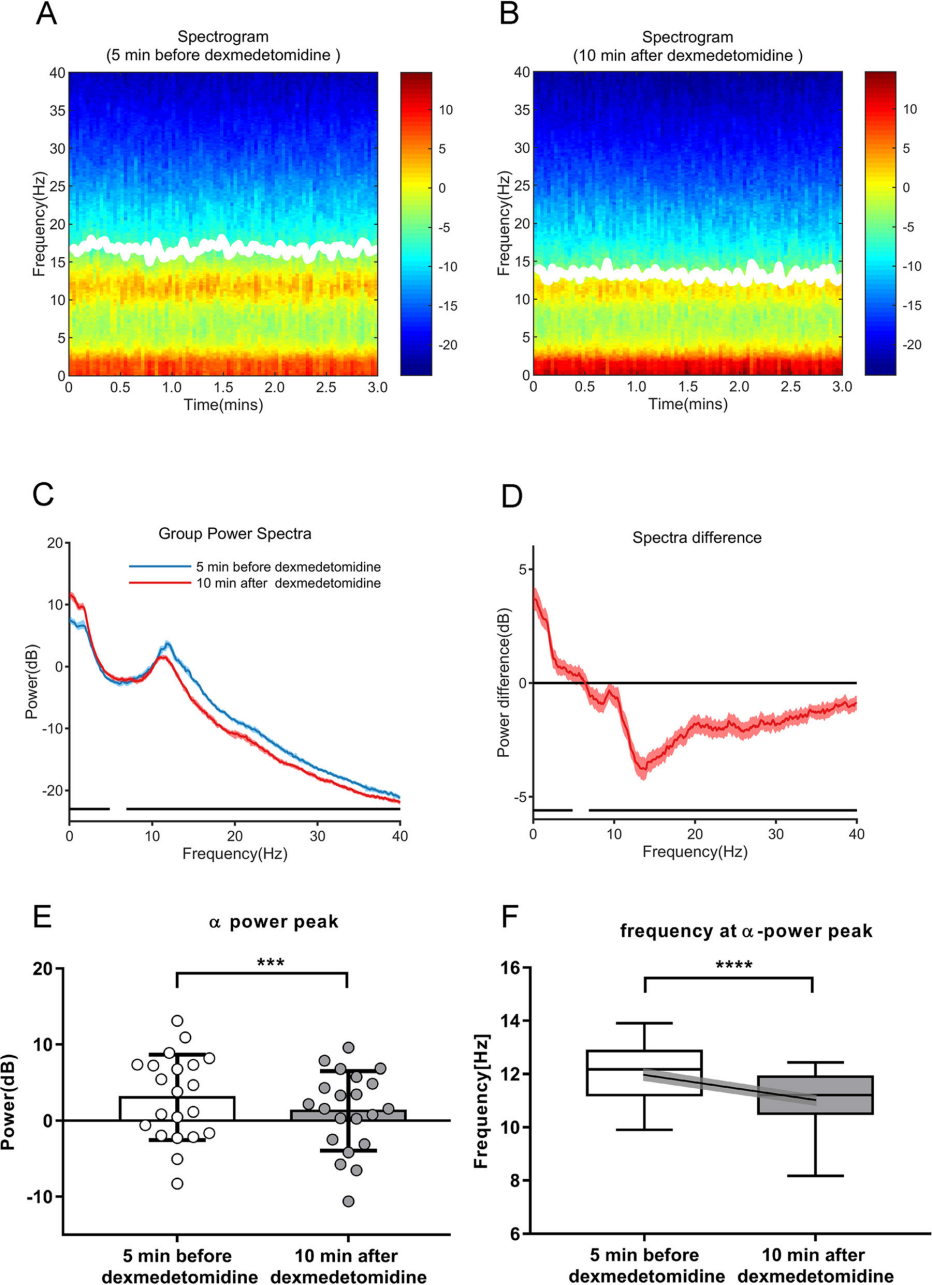
Power spectral analysis and comparison of group-level spectrograms. **A** Group-level median frontal spectrogram showing prominent power in the slow wave and alpha band ranges. **B** Group-level median frontal spectrogram showing a distinct decrease in power in the *α* frequency band. White solid lines in **A** and **B** represent SEF95. **C** Medians of 21 superimposed power spectra from 5 min before Dex injection (blue line) versus 10 min after (red line). From the pre- to the post-injection time points, the *α* peak showed a power reduction and slight left-shifting of frequency, while the power of slow waves increased sharply. **D** Median spectral power difference curve across the two periods at each frequency; shading represents 95% CI range. Approximately 10 min after Dex injection, EEG power was greater in the range of 0~4.8 Hz and lesser in the range of 6.8~40 Hz. The horizontal lines above the *x*-axis in **C** and **D** represent the frequency range at which the power differed significantly across evaluation time periods. **E** Comparison of mean *α* peaks (± SDs) before versus after Dex injection for 21 cases; ***p < 0.001, paired *t* test. **F** Comparison of frequencies of *α* peaks plotted as box plots. Horizontal lines in the boxes indicate the median values, the box top/bottom indicates 25% and 75% quartiles, and whiskers indicate the minimum and maximum values for the 21 cases. The means are shown with the end points of the solid diagonal line, the shading around which reflects standard errors of the means; ****p < 0.0001, paired *t* test

Figure 2 shows the comparison of superimposed spectrograms and power spectra for the 21 cases 5 min before and 10 min after Dex injection. The group-level power spectrogram analysis revealed distinct reductions in the power and frequency of the *α* frequency band, along with an increase in slow waves power from the pre-Dex injection period to the post-injection period (Fig. 2A, B). Accurately, electroencephalogram power was increased in the 0–4.8 Hz frequency range and decreased in the 6.8–40.0 Hz range (Fig. 2C, D, *p* < 0.05, bootstrap). The mean power (Fig. 2E, mean (95% CI), *p* < 0.001) and median frequency (Fig. 2F, median (25th, 75th percentiles), *p* < 0.0001) of *α* oscillation peak values were significantly reduced from 3.1 dB (0.5 to 5.6 dB) and 12.2 Hz (11.2 Hz, 12.9 Hz) prior to Dex injection to 1.3 dB (− 1.1 to 3.7 dB) and 11.2 Hz (10.5 Hz, 11.9 Hz), respectively, after Dex injection, with a bootstrap mean difference(95% CI) of − 1.8 dB (− 5.0 to 1.5 dB) and bootstrap median difference (95% CI) of − 1.0 Hz (− 1.5 to 0 Hz). The median power (Fig. 2C, *p* < 0.05) of slow oscillation values (*δ*-*θ* waves) was increased from 4.5 dB (4.4 dB, 4.5 dB) prior to Dex injection to 7.7 dB (7.7 dB, 7.8 dB) after Dex injection, with a bootstrap median difference of 3.3 dB (3.1 to 3.4 dB).

**Fig. 3:**
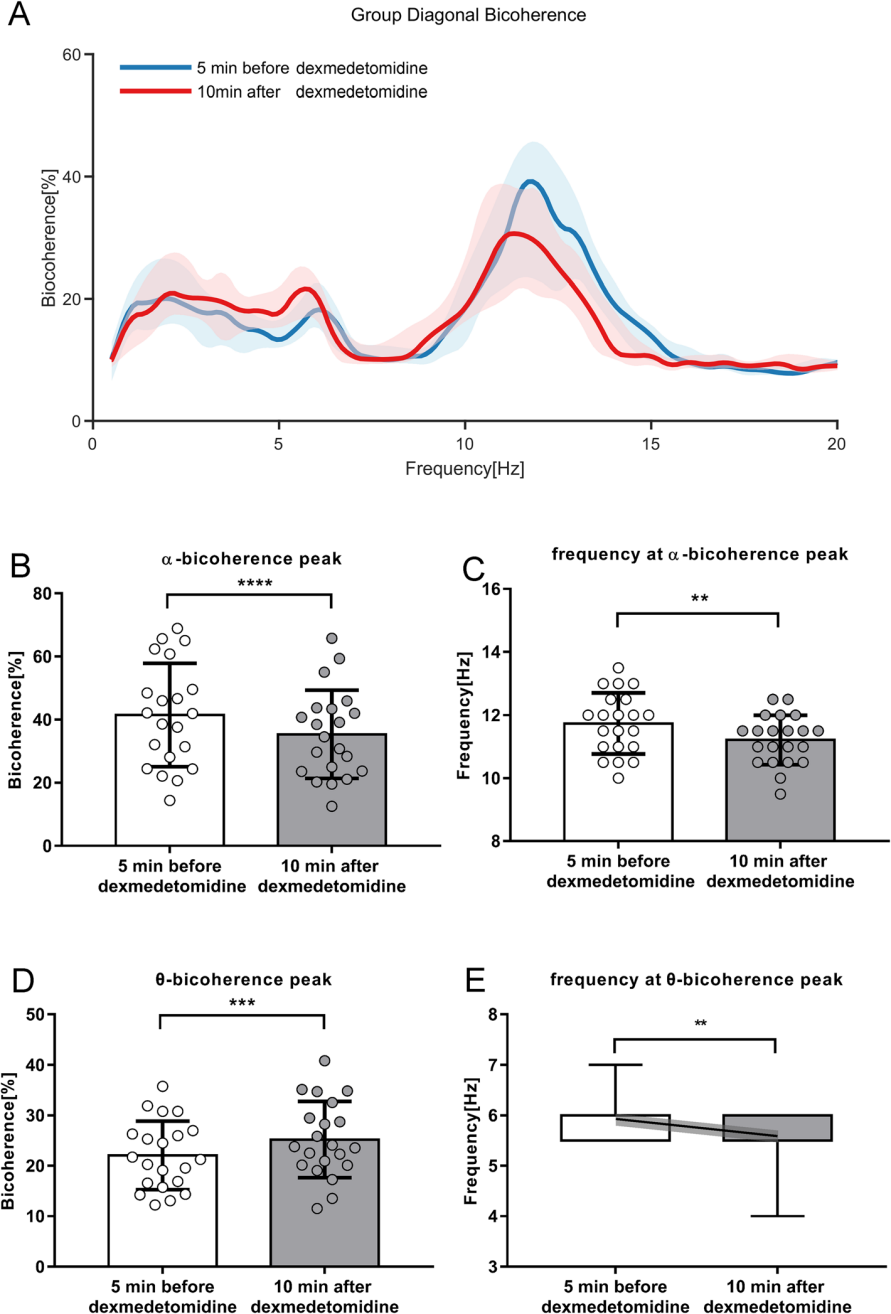
Comparison of group-level diagonal bicoherence 5 min before and 10 min after Dex injection. **A** Median curve of superimposed diagonal bicoherence spectra from 21 cases 5 min before (blue line) and 10 min after (red line) Dex injection, shown with 95% CIs. From the pre- to the post-injection data, bicoherence of *α* peak values decreased while bicoherence of slow *θ* waves increased. **B** Mean α-bicoherence peaks of 21 cases; error bars are SDs; ****p < 0.0001, paired *t* test. **C** Mean frequency of α-bicoherence peaks of 21 cases; error bars are SDs; **p < 0.01, paired *t* test. **D** Mean θ-bicoherence peaks of 21 cases; error bars are SDs; ***p < 0.001, paired *t* test. **E** Frequency of *θ*-bicoherence peaks, box top/bottom indicates the 25% and 75% quartiles, and error bars indicate the minimum and maximum among the 21 cases. Median values are superimposed on the 75th and 25th quartile values. The means are shown with the end points of the solid diagonal line, the shading around which reflects standard errors of the means; **p < 0.01, Wilcoxon signed-rank test

Figure 3 shows the comparison of group-level diagonal bicoherence and *α*&*θ* bicoherence peaks 5 min before and 10 min after Dex injection. The superimposed EEG results for the 21 cases included in our diagonal bicoherence spectra analysis exhibited a similar trend (Fig. 3A) compared to group-level spectra. The mean bicoherence (Fig. 3B, *p* < 0.0001) and frequency (Fig. 3C *p* < 0.01) of *α* oscillation peak values were decreased dramatically from 41.5% (34.0 to 49.0%) and 11.7 Hz (11.3 to 12.2 Hz) 5 min before Dex injection to 35.4% (29.0 to 41.8%) and 11.2 Hz (10.9 to 11.6 Hz) 10 min after Dex injection, respectively, with bootstrap mean differences of − 6.1% (− 14.9 to 2.8%) and − 0.5 Hz (− 1.0 Hz to 0 Hz), respectively. Meanwhile, the mean bicoherence of slow *θ* oscillations peak values was augmented dramatically (Fig. 3D *p* < 0.001) from 22.1% (19.0 to 25.2%) to 25.2% (21.8 to 28.6%), with a bootstrap mean difference of 3.1% (− 1.1 to 7.4%), accompanied by a slight diminishment of frequency (Fig. 3E, *p* < 0.01) from 6.0 Hz (5.5 Hz, 6.0 Hz) before Dex injection to 5.5 Hz (5.5 Hz, 6.0 Hz) after Dex injection, with a bootstrap median difference of − 0.5 Hz (− 0.5 to 0.5 Hz).

**Fig. 4:**
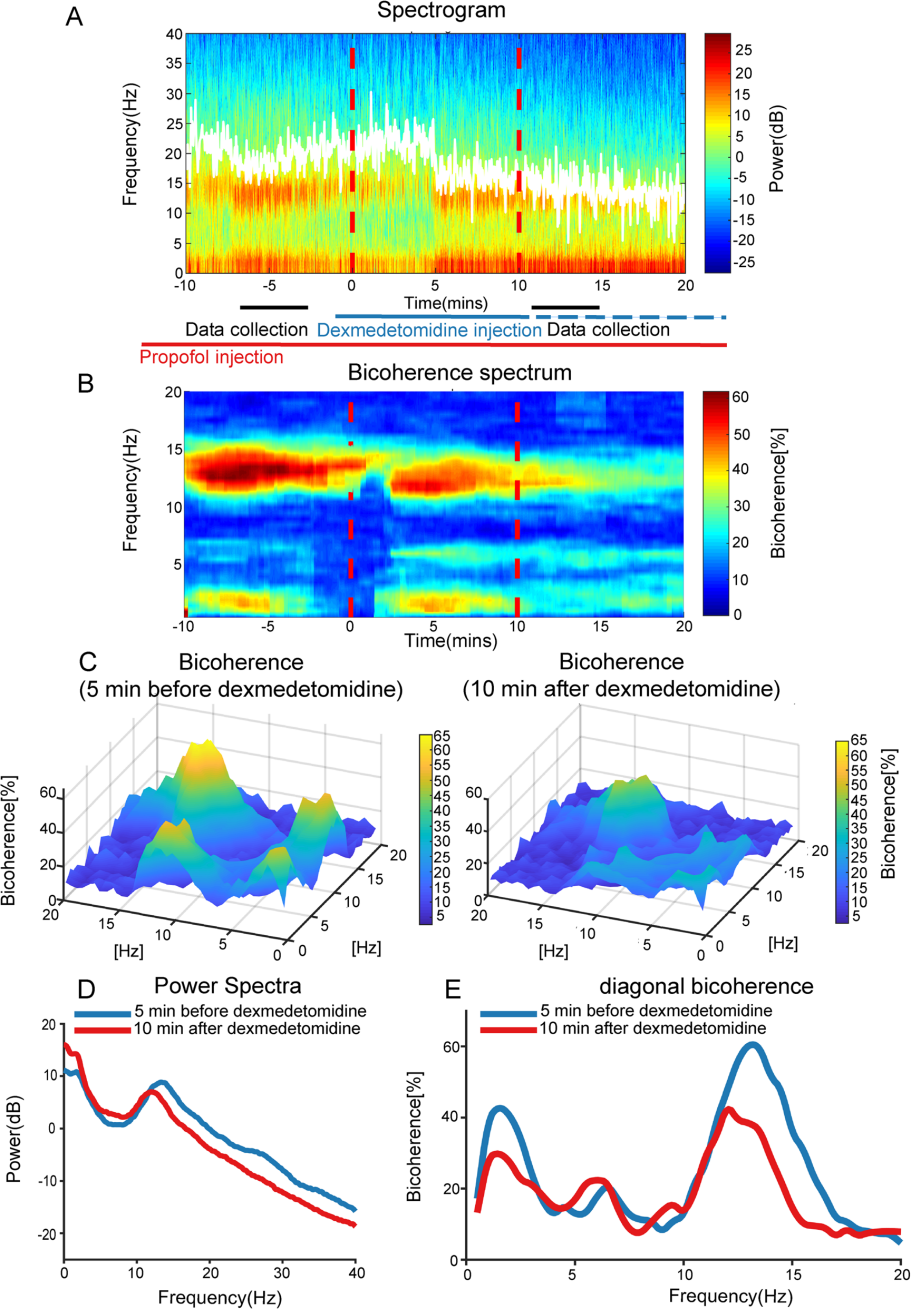
Representative time course of a spectrogram and bicoherence spectrum of a patient under propofol anesthesia supplemented with Dex. **A** Frontal cortical time-frequency spectrogram of the Fp1 channel during anesthesia. **B** Frontal cortical bicoherence spectrum during anesthesia. **C** Bicoherence 5 min before injection (left) and 10 min after injection (right) of Dex for all pairs of frequencies. **D** Frontal power spectra 5 min before (blue line) and 10 min after (red line) Dex injection. Note that *α* peak power and frequency decreased from the former to the latter time period. **E** Diagonal bicoherence of 5 min before (blue line) and 10 min after (red line) Dex injection. Note that the *α* bicoherence peak, *α* peak frequency, and slow *θ* peak frequency decreased while the slow *θ* bicoherence peak increased from the former period to the latter period

The aforementioned changes in power and diagonal bicoherence spectra can be readily observed in the representative case shown in Fig 4, which illustrates a pattern consistent with our group-level results. Note that the time-related power and bicoherence spectra from this case (Fig. 4A, B) are divided into components delineating the 5-min pre-injection and 10-min post-injection evaluation periods to allow intuitive visualization of the relationships among frequency, power, and bicoherence. Several minutes after Dex injection, gradual changes in the electroencephalogram become apparent (Fig. 4D, E), with increased power and bicoherence of slow *θ* oscillations. Meanwhile, the decreased power and bicoherence of *α* oscillation peak can be seen accompanied by diminution of the frequency band corresponding to the *α* peak in both the power and bicoherence spectra.

## Discussion

The findings of the present study showed that intravenous Dex injection induced a deepening of anesthesia in patients under propofol GA, as evidenced firstly by reductions in PSI and SEF95 values, consistent with the previous results (Araki et al., 2018; Hayashi et al., 2008), and secondly by increases in the slow waves power and *θ* oscillation bicoherence peaks. Conversely, the Dex injection resulted in decreases in *α* oscillation peak power and bicoherence, accompanied by decreased *α* peak frequencies in the power and bicoherence spectra. We believe this EEG pattern change reflects a primary drug effect on a complex neural network system through multiple mechanisms.

### Neural mechanisms behind *α* oscillation power and frequency changes

Previous studies have documented *α* oscillation changes that are characteristic of propofol and Dex separately. Xi et al. (Xi et al., 2018) demonstrated hierarchical changes in propofol-induced effects, including decreases in occipital *α* oscillations during moderate sedation and propofol-induced *α* anteriorization during loss of consciousness. Meanwhile, Dex has been shown to attenuate *α* oscillation power across the frontal-posterior cortex (Bhattacharya et al., 2014; Cantero et al., 2002; Cantero et al., 1999). Dex suppression of NE release from the LC reduces the hyperpolarization-activated current *I*_*h*_ (Lee & McCormick, 1996) and leads to attenuated *α* oscillations. Power spectral analysis of EEG data reveals changes in peak amplitudes within frequency bands that are consequent to increased or decreased numbers of active neurons firing in synchrony (Fingelkurts et al., 2006). Thus, Dex attenuation of *α* oscillations could reflect reduced synchronization of thalamic reticular neurons.

Interestingly, we observed another conspicuous Dex-induced phenomenon. That is, the *α* peaks in both the power and bicoherence spectra shifted, in tandem, to a lower frequency following Dex injection. Upon weakening of a rhythm formed in the thalamocortical reverberating network, the peak value of the corresponding frequency of the power spectrum will be reduced because the change in the reverberation signal will attenuate the number of active neurons in the reentry loop. The bicoherence peak of the rhythm will be attenuated because of the phase coupling caused by the reverberation waves. Although the precise mechanisms underlying diminution of *α* frequency oscillations are not known, these EEG changes are likely to be induced by the modulation of the thalamocortical relay (TC)-reticular neuron activation dynamics in thalamocortical reverberant loops. It is worth noting that the frequencies of the *α* oscillations that were reduced by Dex in both the power and bicoherence spectra in our study are similar to the spindle oscillation frequency range (12~16 Hz) evident in both non-rapid eye movement stage II sleep and sedation induced by Dex (alone). Spindle oscillations have been observed in both propofol- and Dex-induced sedation (Huupponen et al., 2008; Ozgoren et al., 2010). Dex-induced spindle oscillations have been reported to show conspicuous increases during moderate sedation but attenuation during deep sedation. Meanwhile, propofol-induced spindle activity was found to be increased from frontal to posterior areas during moderate sedation and to be dramatically increased in frontal regions during deep sedation (Xi et al., 2018). Differential spindle oscillation characteristics under the influence of propofol versus Dex during wakefulness and moderate sedation suggest that these two drugs have distinct actions on thalamocortical sedation mechanisms. Frontal *α* wave frequency reductions may reflect the restriction of communication within frontal thalamocortical circuits from a wide frequency band to a narrow one, thus promoting a change in consciousness (Ching et al., 2010; Supp et al., 2011). We hypothesize that the frequency shifts from *α* oscillations to spindle-like waves may reflect the maintained dominance of the propofol action pathway after Dex injection at the doses used in our study. Although the mechanisms underlying it are not clear, decreases in *α* frequency power reflect a deepening of anesthesia caused by the Dex injection in patients under propofol GA.

### Neural mechanisms behind the change of bicoherence changes

Coherence is often used to compare the similarity of two signals, as well as to detect wave synchronizations. Coherence analyses may also provide insights into the internal relationships among specific cortical areas. However, because thalamocortical reverberant features produce massive EEG synchronizations (not of a linear structure), they cannot be examined by analyzing the coherence between two defined cortical signals. Bicoherence, an effective method to detect the nonlinear reverberation characteristics of the thalamic cortex, can be used to assess the correlation regulation between the thalamic cortex and the cortical regions. Indeed, the PSI value, which is calculated from bispectral analysis (Drover & Ortega, 2006; Prichep et al., 2004), may itself be related to bicoherence. Because the thalamocortical network represents a critical factor in anesthesia, its activities are prominent in systematic and qualitative studies of bicoherence changes across different anesthetic depths. In a nonlinear reverberation system, the reverberation circuit’s output signal is expected to reenter the system as an input signal and thus lead to self-modulation characteristics, namely the components of intermodulation products (i.e., signal components generated by multiplying input signal components). Bicoherence tends to increase in these frequency components owing to quadratic phase coupling between input signal components. Consistent with Hayashi et al.’s (Hayashi et al., 2008) suggestion that bicoherence of certain EEG waves should change with the deepening of anesthesia, we found that deeper anesthesia was associated with increased slow wave bicoherence together with reduced *α*-wave bicoherence, consistent with previous reports (Hagihira et al., 2002; Hayashi et al., 2008; Morimoto et al., 2006).

### The thalamocortical network may be the dominant factor of EEG changes

In patients under the influence of classical GABAergic GA agents, such as sevoflurane and propofol, *α* oscillation bicoherence is augmented and the *α* bicoherence peak frequency exhibits a downward shift following intravenous injection of droperidol; these changes are consistent with deepening of anesthesia from a light to a moderate level. Our present finding of Dex injection-induced reduction in *α*-oscillation bicoherence is consistent with a deepening of anesthesia beyond a moderate level (Xi et al., 2018; Murphy et al., 2011). Therefore, we suggest that in the progressive course of deepening of the anesthesia state, the bicoherence and power of slow waves increases, the mean frequency of *α* waves decreases, and the bicoherence of *α* waves increases until a moderate level of anesthesia is reached, after which *α* waves decrease with further deepening of the anesthesia level. These changes may reflect the electrophysiologic activity of reticular and thalamocortical relay neurons. Thalamic reticular neurons drive the spindling *α* rhythm of early sleep (Hayashi et al., 2008; Destexhe et al., 1993), and thalamocortical relay neurons drive the slower *δ*-*θ* rhythms of deeper sleep (Hayashi et al., 2008; Destexhe et al., 1993). Both rhythms are controlled via intrinsic and synaptic activities within the thalamocortical reverberating network (Sohal et al., 2006; Yousif & Denham, 2005). The conductance of one cell type among reticular and thalamocortical relay neurons might underlie the predominance of *δ*-*θ* bicoherence, which may alter the intrinsic and synaptic electrophysiologic properties resulting in the shift from spindling *α* waves to a *δ*-*θ* rhythm. These results suggest that thalamocortical relay neurons, rather than reticular neurons, have a principal role in regulating EEG rhythms in the thalamocortical network and deepening of anesthesia by Dex injection.

A limitation of this study is that propofol was not injected in a target-controlled manner, thereby allowing possible fluctuations in blood concentrations of propofol, which may affect EEG data stability. However, our SEF95 and PSI data, together with our EEG data, indicate that the anesthesia states of the patients were relatively stable. It is worth noting that although propofol-Dex anesthesia has been widely used in clinical settings, ~ 10% of the patients in this study had episodes of bradycardia during their operations, which could be suppressed with the muscarinic receptor antagonist atropine. Furthermore, in this study, we monitored and analyzed only four-channel frontal EEG data, we could not detect the propofol-associated alpha anteriorization phenomenon (Akeju et al., 2014a; Murphy et al., 2011), which remains to be clarified. Theoretical analyses of the noteworthy EEG changes described herein, which have been discussed based on previously clarified electrophysiological knowledge, will be instrumental in determining correlative pathways and networks.

## Conclusions

The selective α2-adrenoceptor agonist Dex augmented the slow waves power and *θ* oscillation bicoherence peak. Following Dex injection, the frequency corresponding to the *α* peak was reduced in both power and bicoherence spectra, and these changes were accompanied by reductions in the power and bicoherence of the *α* peak. These profound EEG changes suggest that Dex enhances the effects of propofol anesthesia, moving patients from a moderate to a deep state of anesthesia. These findings may provide a theoretical basis and reference for drug administration protocols aimed at reducing the dosage of anesthetics while maintaining sufficient depth of anesthesia. Drug-specific electroencephalogram signatures should be explored in-depth to elucidate molecular and neural circuit mechanisms of anesthetic actions.

## Supplementary Information

**Additional file 1: Figure S1.** STROBE diagram.

## Data Availability

The datasets used and/or analyzed during the current study are available from the corresponding author on reasonable request.
