# Supplementary material for: Electroencephalography-demonstrated mechanisms of dexmedetomidine-mediated deepening of propofol anesthesia: an observational study
Source: Perioper Med (Lond). 2021 Dec 9;10:44. doi: 10.1186/s13741-021-00213-4 (PMC8656083; doi:10.1186/s13741-021-00213-4)
Supplement: Supplementary file 1 — Additional file 1: Figure S1. STROBE diagram. [file 13741_2021_213_MOESM1_ESM.docx]

EEG Analysed (n= 21 )
♦ Excluded from analysis (give reasons) (n= 0)

5 min before and 10 min after DEX EEG(n=24)

♦ Received allocated intervention (n= 24 )

♦ Did not receive allocated intervention (give reasons) (n= 0 )

Excluded (n= 3)

♦  due to poor quality EEG (n = 3)

Excluded (n= 2)

♦  due to bad channels (n = 2)

Assessed for eligibility (n= 26 )

10 min after

DEX Group

(n=21)

5 min before

DEX Group

(n=21)

Fig S1. Strengthening the Reporting of Observational Studies in Epidemiology(STROBE)diagram.

DEX , dexmedetomidine .
